# Monitoring Brown Bears in Kazakhstan: A Pilot Study from the Altai Mountain Region

**DOI:** 10.1002/ece3.73272

**Published:** 2026-03-18

**Authors:** Sanzhar Kantarbayev, Aimee Tallian, Matthew Grainger, Brett K. Sandercock, Alexander Kopatz

**Affiliations:** ^1^ Laboratory of Biocenology and Game Management Institute of Zoology of the Republic of Kazakhstan Almaty Kazakhstan; ^2^ Norwegian Institute for Nature Research Trondheim Norway

**Keywords:** Altai mountains, camera trap, Central Asia, Eastern Kazakhstan, large carnivore monitoring, ungulates, *Ursus arctos*

## Abstract

Monitoring populations of large carnivores that are often elusive and occur at low densities is intrinsically difficult. The challenges are often exacerbated in developing nations with less infrastructure or resources for wildlife surveys. In regions where little is known about wildlife populations, even basic information about the presence of large carnivores in a landscape is an important first step towards implementing sustainable management practices to promote co‐existence with humans. Here, we conducted a first‐ever pilot study in the Altai Mountain Region of Kazakhstan using camera traps to assess the presence of brown bear ( *Ursus arctos* ) and their primary prey species in the study region, and to better understand potential drivers of variation in activity. Between 2019 and 2023, we deployed 10 camera traps which collected data for a total of 4654 days. Bear activity varied seasonally; all activity occurred during the ‘active season’ with activity peaking in May/June. Camera traps also detected 13 other species including a range of potential ungulate prey and other carnivores. Our study was conducted at a relatively local scale, but represents a first step towards understanding the presence of brown bears and other mammals in a remote region of central Asia. There is currently limited scientific understanding of where bears occur in the Altai Mountains of Kazakhstan or the neighboring countries of China, Mongolia, and Russia. Our results are therefore useful for the adaptive management of brown bears at the local scale, and the lessons learned from our study can be used to scale up and optimize wildlife monitoring efforts by local managers and researchers in Kazakhstan.

## Graphical abstract

Here, we present results from the first camera trapping operation designed to detect brown bears in the Altai Mountain Region of Kazahstan and highlight some lessons learned from the pilot study. Our study was conducted at a relatively local scale but represents a first step towards understanding the presence of brown bears and other mammals in a remote region of Central Asia.

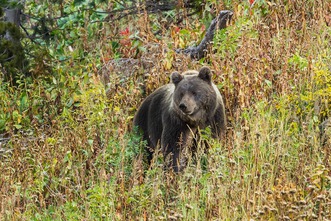

## 1 Introduction

Populations of large carnivores have recovered in Eurasia and other parts of the world (Chapron et al. 2014), but many also remain severely threatened, largely due to a combination of environmental change, habitat loss, prey depletion, and persecution resulting from human‐wildlife conflicts (Ripple et al. 2014). Large carnivores play an important role in ecosystem function via their top‐down effects on prey populations (Terborgh et al. 2001; Wolf and Ripple 2018). Understanding which carnivore species are present in a landscape, and quantifying their population numbers, density, and behavioral patterns are therefore fundamental goals of wildlife researchers, managers, and conservationists worldwide.

Monitoring large carnivore populations is intrinsically difficult, however, because many species have secretive behavior, occur at low densities, and range over large areas (Kendall et al. 1992; Linnell et al. 1998; Thompson 2013). Furthermore, the challenges associated with large carnivore monitoring are often exacerbated in developing countries with limited infrastructure, because wildlife surveys are often time‐intensive and require sufficient funding and expertise (Braczkowski et al. 2023). Yet, monitoring these populations is particularly important as large carnivores are often poorly tolerated and subject to both legal management and illegal persecution, which can result in abrupt changes in population numbers if left unchecked (Ripple et al. 2014). Thus, in areas where little is known about these populations, even a basic understanding of where large carnivores are present in a landscape is an important first step towards implementing sustainable management practices.

As an “omnivorous” large carnivore, brown bears ( *Ursus arctos* ) represent unique management and conservation challenges. Not only are bears capable of injuring humans and depredating livestock (Bautista et al. 2017; Bombieri et al. 2019), but their omnivorous diet and propensity to scavenge bring them into a wide range of other conflicts with humans. Bears regularly feed on and destroy apiaries, damage fruit and tuber crops, scavenge garbage, and destroy other human property such as sheds, barns, and bins (Can et al. 2014; Bautista et al. 2017). While our understanding of the biology and ecology of brown bears, including their population dynamics, has improved over the last several decades in Europe and North America, little is known about the status of brown bear populations in mid‐east and central Asia, including Kazakhstan (McLellan et al. 2016).

**FIGURE 1:**
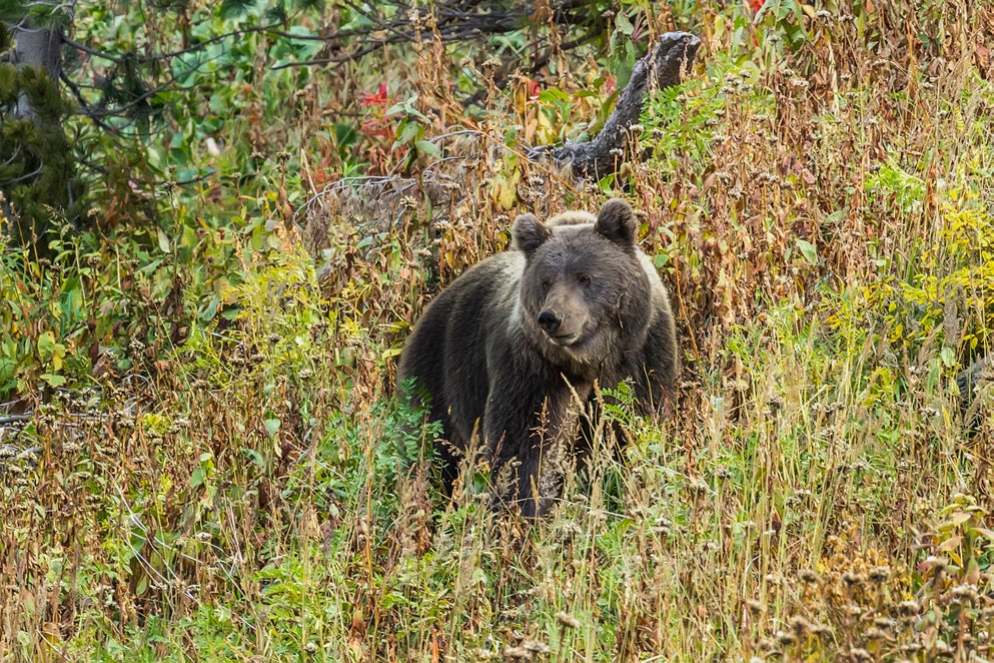
East Siberian brown bear ( *Ursus arctos collaris* ) in the Altai Mountain Range in northeastern Kazakhstan. Photo by Alexander Klimenko.

Kazakhstan supports two subspecies of brown bear in different regions of the country: the Tien Shan brown bear (*U.a. isabellinus*) in the Tien Shan Mountain Range in the southeast and the East Siberian brown bear (*U.a. collaris*) in the Altai Mountain Range in the northeast. (Figure 1). Bear populations are managed differently in the two regions; the Tien Shan brown bear is considered threatened, whereas the East Siberian brown bear is considered a game species. The available literature contains reliable, but limited information on aspects of the species' ecology (Grachev 1972; Lobachev 1972; Baydavletov 1993, 2010; Zinchenco 2006), but little is known about the brown bear populations in Kazakhstan. As of yet, there have been no full‐fledged systematic studies aimed at understanding how many brown bears are in Kazakhstan or even what areas they occupy within the broad mountain ranges of their extant range.

Here, we conducted a first‐ever pilot study in the Altai Mountain Region of Kazakhstan to assess the use of camera traps for detecting and monitoring brown bears and their associated community of carnivores and ungulates. We selected the Altai Mountain Region as brown bear densities were likely higher than in the Tien Shen Mountain Range, even though the Altai population is likely negatively affected by local development. Development of mining, agriculture, and tourism, and the consumption of local biological resources in the mountain areas of the Eastern Kazakhstan region during the past 40–50 years have turned the region into one of the most developed industrial areas of the Republic (Bureau of National Statistics of the Agency for Strategic Planning and Reforms of the Republic of Kazakhstan, 2024). The country's largest mining and metallurgical enterprises producing non‐ferrous and rare metals are located here. The scale of technogenic impact has been substantial and has led to sustained changes in the environment, which can potentially have a significant negative impact on local ecosystems (Wang and Dai 2020). The region is also subject to high rates of human‐bear conflict, and conflicts between humans and bears have become more frequent in recent years. Conflicts have mainly involved beekeepers and farmers which frequently result in illegal killing of bears. In a 3 year period between 2019 and 2021, more than 30 cases of bear killing were recorded (Kantarbayaev 2019). The underlying reasons for the increased conflict remain unclear but could be due to increasing numbers of brown bears in the Eastern Kazakhstan region, increased anthropogenic impact on brown bear habitats, or changes in human–bear interactions without appropriate management solutions.

A better scientific understanding of where bears occur in the Altai Mountains of Kazakhstan is urgently needed to develop adaptive local management and conservation strategies. We therefore deployed camera traps, a comparatively inexpensive tool for passive monitoring, to explore their potential to detect bears in the region. We kept the trial study area constrained to balance trade‐off between effort and quality of data, because resources were limited and remote areas were difficult to access in the rugged terrain of the region. We then quantified camera trap detections of bears and three prey species of ungulates that represent potential food resources: moose ( *Alces alces* ), maral or Caspian red deer (*Cervus canadensis sibiricus*), and Siberian roe deer ( *Capreolus pygargus* ). We used continuous monitoring to explore how detection rates varied seasonally and diurnally, and to explore how detection rates of bears were related to proximity to roads, villages, and other human infrastructure. Our results provide a valuable baseline for management of brown bears at a local scale. Moreover, the lessons learned from this study can be used to scale up and optimize monitoring efforts for bears and other wildlife by local managers and researchers within Kazakhstan who will contribute foundational data to international biodiversity monitoring initiatives.

## 2 Methods

### 2.1 Study Area

**FIGURE 2:**
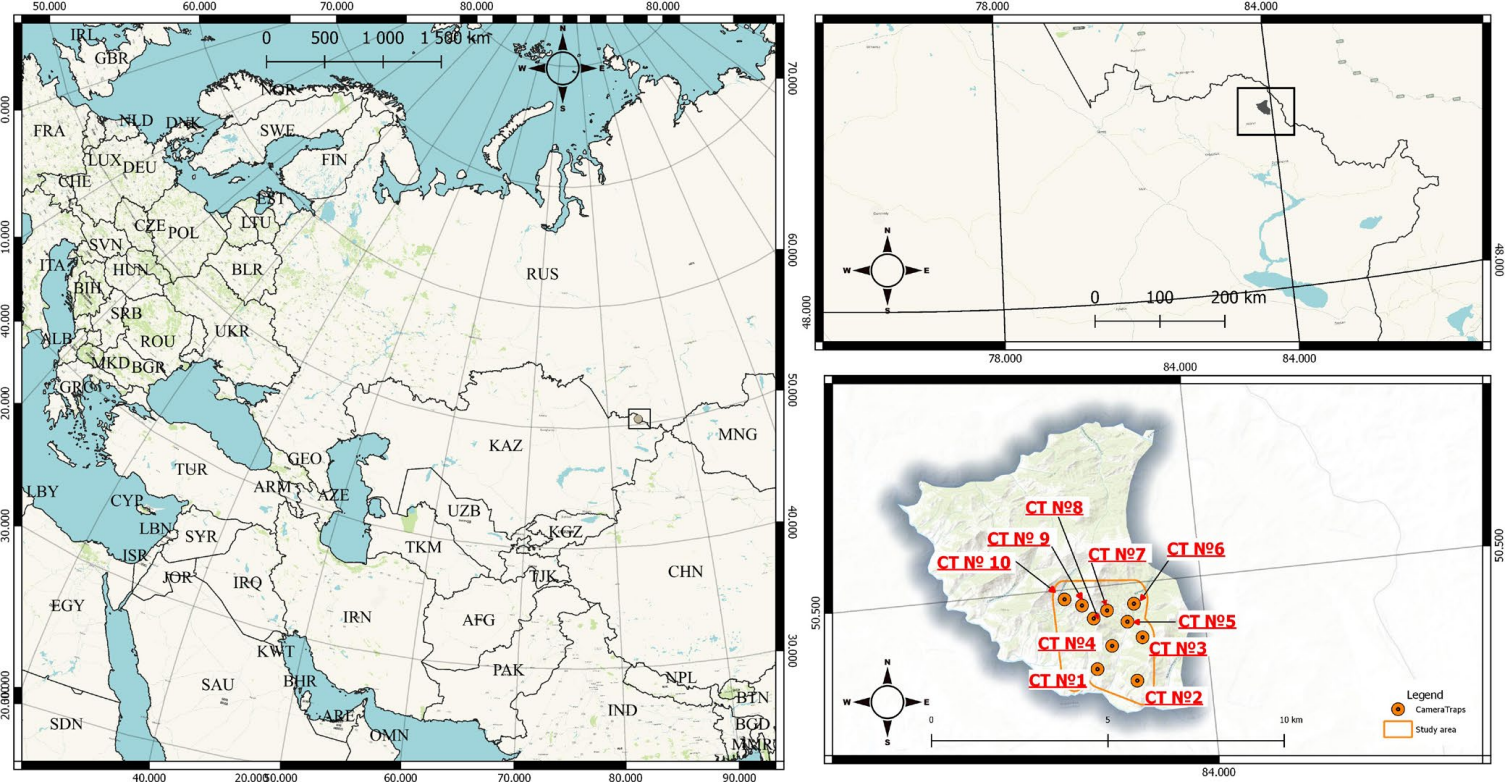
Study region (black box) in the northern part of the Altai Mountain Region in northeastern Kazakhstan (left and upper right panels) and the main study area highlighted in orange (lower right panel). Camera traps (red circles) are listed in numerical order (Table 1).

The study was conducted in the Altai Mountain Region in northeastern Kazakhstan (elevation 300–4506 m), where the range of the east Siberian brown bear population spans an area of 36,500 km^2^ (Figure 2). The study area was limited to a small section of the northern part of the Altai, hereafter referred to as “Western Altai” (12,608 km^2^, elevation 500–2776 m). The main habitats are mixed forests of cedar ( *Pinus sibirica* ) and cedar‐fir (*Abies sibirica*) which grow along river valleys and mountain slopes (430–1900 m). Subalpine meadows are found at higher elevations above ~1900 m, and are classified as either high or low grass systems. The meadows are interspersed with sparse forests of cedar and larch ( *Larix sibirica* ) with thickets of shrubby willows (*Salix arbuscula*) and birches (*Betula fruticosa*). Alpine meadows do not have clear elevational boundaries. The climate in the Altai Mountain Region is characterized by a continental climate with strong seasonality that is also affected by the mountainous terrain, resulting in hot dry summers and cold snowy winters. The mean annual air temperature in the middle mountains is about −3°C, with a mean total annual precipitation of 630 mm. In the highlands, the mean annual air temperature is −8°C, with a mean total annual precipitation of 1500 mm. Summers are warm with daytime temperatures averaging 20°C–23°C and nighttime temperatures down to 7°C–9°C, and summer precipitation (9–12 days per month) occurs in the form of short heavy rains. Night frosts begin to occur in the second half of September, with daytime temperatures ranging from 5°C to 15°C, whereas night temperatures range from +1 to −5°C. Stable snow cover usually begins in the middle of November and continues until late March/early April when snow begins to melt. The accumulated winter snowpack is usually completely gone from valleys and in intermountain depressions by mid‐April and in the mountains in early June.

The brown bear appears to primarily occur in the mountain forests of the Altai Mountain Region, ranging from the foothill forests to the alpine meadows of the highlands. The only previous survey of brown bears in the region suggested there could be about 2400 brown bears in the Kazak part of the Altai Mountains Region, but the survey was non‐systematic and the estimate is considered unreliable (Jingfors 2016). Brown bears in the region are a game species, with legal hunting occurring between 15 April and 15 May in the spring and 1 September to 31 December in the autumn, but they are also likely subject to losses from persecution and illegal hunting. Little is known about the wildlife community in this region, but local knowledge suggests that potential ungulate prey species in the area include moose, maral or Caspian red deer, and Siberian roe deer, as well as wild boar ( *Sus scrofa* ) and Siberian musk deer ( *Moschus moschiferus* ). Other species of carnivores that could be potential competitors for prey, or benefit from scavenging opportunities, include wolves ( *Canis lupus* ), wolverine ( *Gulo gulo* ), lynx ( *Lynx lynx* ), red fox ( *Vulpes vulpes* ), Asian badger ( *Meles leucurus* ), and sable ( *Martes zibellina* ). Little is known about the population size and distribution of these species in this region.

### 2.2 Data Collection

Brown bear surveys were conducted in a 5 year period between 2019 and 2023 using remotely triggered camera traps (Bushnell Trophy Cam HD and Bushnell Trophy Cam HD Aggressor) (Table 1). Ten camera traps were deployed in the ‘Kuchikha’ hunting estate (15 km^2^; Figure 2) under a scientific cooperation agreement dated January 30, 2019, and renewed on April 12, 2022. Cameras were attached to trees at a height of 80 cm, which is the average height at the withers of the Altai brown bear. Cameras were set up with a focal angle slightly downwards to better observe young if present and to optimize detections of bear activity while minimizing possible interference from vegetation or other obstructions. The camera traps were deployed at forest sites where bear sign had previously been detected and then left to collect data year‐round when possible to determine the earliest and latest appearance of bears outside of the winter period when bears were hibernating in dens. We also calculated the distance (km) to the nearest road and to the nearest village from each camera trap as an index of proximity to potential anthropogenic disturbance.

**TABLE 1:** Information for all cameras deployed in the Altai Mountain Region study area in northeastern Kazakhstan, including camera number, location name, latitude and longitude, elevation (m), distance to road (km), distance to nearest village (km), and the time periods of deployment.

Camera ID	Location name	Lat	Long	Elevation (m)	Dist. road (km)	Dist. village (km)	Time period
KCH01	Kuchiha‐Popov	E83° 51.965′	N50° 26.234′	1164	6.7	4.4	12 Apr–18 Jun, 2019 11 Jul–31 Dec, 2020 1 Jan–9 May, 2021 24 Oct–31 Dec, 2021/1 Jan–21 May, 2022
KCH02	Spruce Ridge	E83° 55.414′	N50° 27.479′	1079	9.1	10	14 Aug–22 Sept, 2019/27 Oct–31 Dec, 2019 1 Jan–31 Dec, 2020 1 Jan–4 May, 2021/11 Sept–22 Sept, 2021
KCH03	Bear Hollow	E83° 54.449′	N50° 28.256′	667	9.2	11	3 Aug–21 Aug, 2019/22 Sept–5 Oct 2019 10 Feb–31 Dec, 2020 1 Jan–10 Jan 2021/25 May–31 Jul, 2021/22 Sept–31 Dec, 2021 1 Jan–31 Dec, 2022 1 Jan–4 May, 2023
KCH04	Sosnovka Pass	E83° 52.064′	N50° 28.560′	666	8	10.9	29 Aug–9 Oct, 2019 31 Jul–3 Oct, 2020
KCH05	Sosnovka Mount	E83° 51.597′	N50° 29.162′	1478	7	9.9	01 Aug–26 Dec, 2020 02 Jan–31 Dec, 2021
KCH06	Vannochki	E83° 51.324′	N50° 29.204′	1478	8.8	12.4	10 Oct–24 Nov, 2019 29 Mar–31 Dec, 2020 1 Jan–5 Jan, 2021
KCH07	Glade Angry Bear	E83° 53.190′	N50° 27.172′	984	6.7	8.7	4 Jul–13 Oct, 2020 4 May–27 Jul, 2021/10 Oct–19 Nov, 2021 12 Feb–3 Mar, 2022/23 Jul–3 Sept, 2022 21 Jan–4 May, 2023
KCH08	Privada	E83° 53.024′	N50° 27.102′	970	6.7	8.7	23 Mar–27 Apr, 2023
KCH09	Solt_lake_larch	E83° 54.012′	N50° 26.187′	1080	6.5	1	4 Jul–10 Oct, 2020/7 Nov–2 Dec, 2020 10 Jan–20 Dec, 2021 1Jan–22 Sept, 2022 21 Jan–08 Feb, 2023
KCH10	Solt Lake Spruce	E83° 54.215′	N50° 27.364′	891	7.9	9.3	13 May–24 Dec, 2021

### 2.3 Data Analysis

We summarized camera trap detections across sites and years to assess species presence and explore seasonal and daily activity patterns of brown bears and their potential prey. For each species, we summed independent detection events per active camera, defining independent events as photographs of the same species that were taken at least 6 h apart, and then used the counts to describe presence and detection frequency. Seasonal trends were assessed by grouping detections by week and plotting weekly totals across the year in relation to the number of active camera traps to control for camera effort. Diel activity patterns were assessed by assigning detections to hourly intervals to visualize the activity distributions. Here, detections were limited to a single detection per species per camera per hour to reduce potential bias from repeated observations. To quantify how elevation and human presence affected brown bear detection, we fitted generalized linear models relating detection frequency (number of detections) to elevation (m), distance to the nearest village (km), and distance to the nearest main road (km), and evaluated model fit using coefficients of determination (*R* ^2^). We fitted generalized linear models with a negative binomial error distribution and log link function to account for overdispersion in count data. Sampling effort (camera‐days) was included as an offset term (log‐transformed). All analyses were conducted with base functions in the statistical programming environment *R* (ver. 4.3.3, R Core Team, 2024) and results were visualized using the package *ggplot* (Wickham 2016).

## 3 Results

We deployed a total of 10 cameras that were placed at a mean elevation of 1046 m (SD = 266.7; range = 666–1478 m). The mean distance to the nearest village was 8.6 km (SD = 3.2; range = 1.0–12.4) whereas the mean distance to the nearest main road was 7.6 km (SD = 1.0; range = 6.5–9.2). In total, the cameras took 7681 pictures of animals, with 901 independent detection records. All animals were unmarked and the same individual could have been detected multiple times during the field study.

**FIGURE 3:**
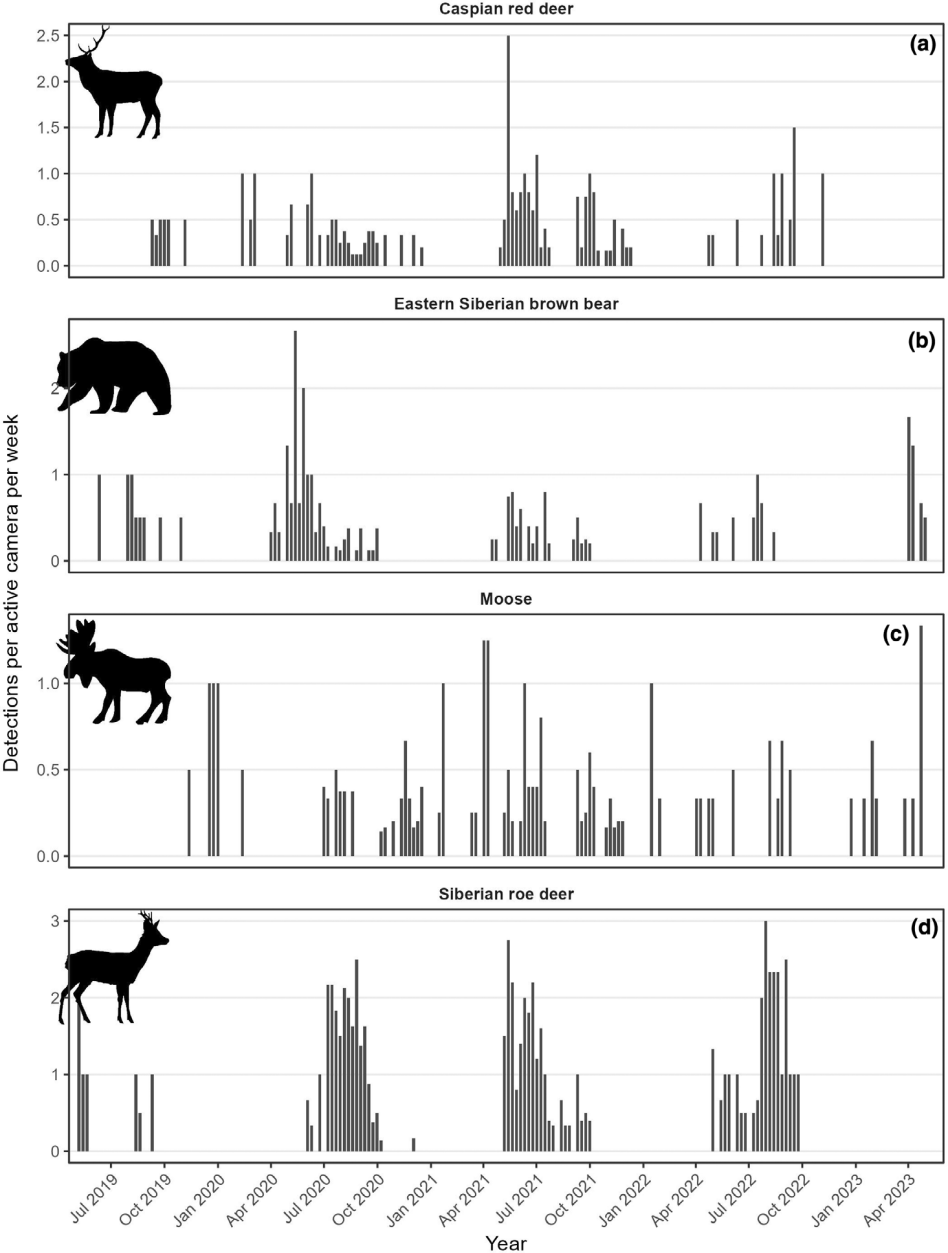
Total number of detections per week per active camera trap for (a) Caspian red deer, (b) Eastern Siberian brown bears, (c) moose, and (d) Siberian roe deer in the Atlai mountains of Kazakhstan, 2019 to 2023.

**FIGURE 4:**
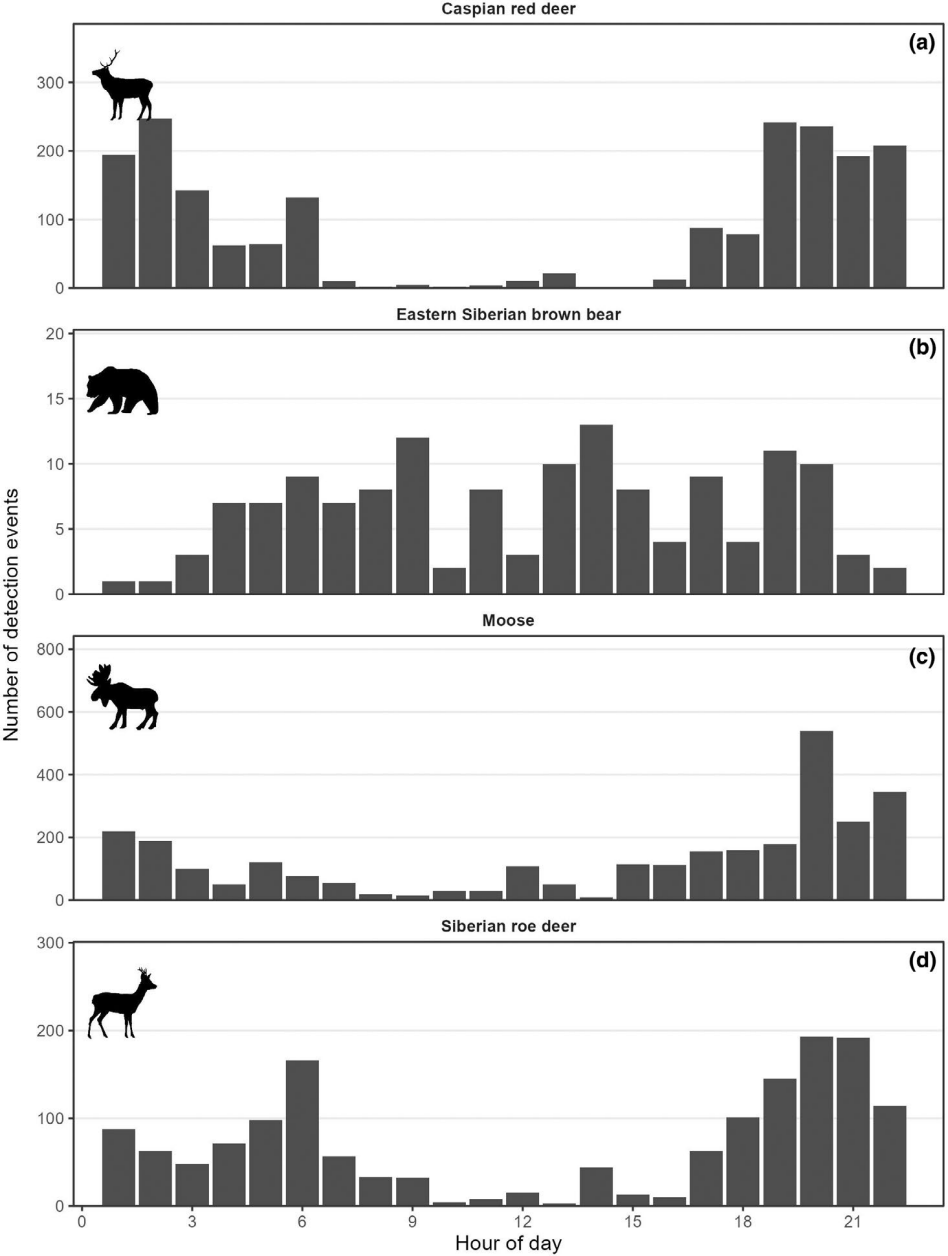
Daily activity patterns of (a) Caspian red deer, (b) Eastern Siberian brown bears, (c) moose, and (d) Siberian roe deer based on the number of detection events per hour across all active camera traps in the Atlai mountains of Kazakhstan, 2019 to 2023.

**FIGURE 5:**
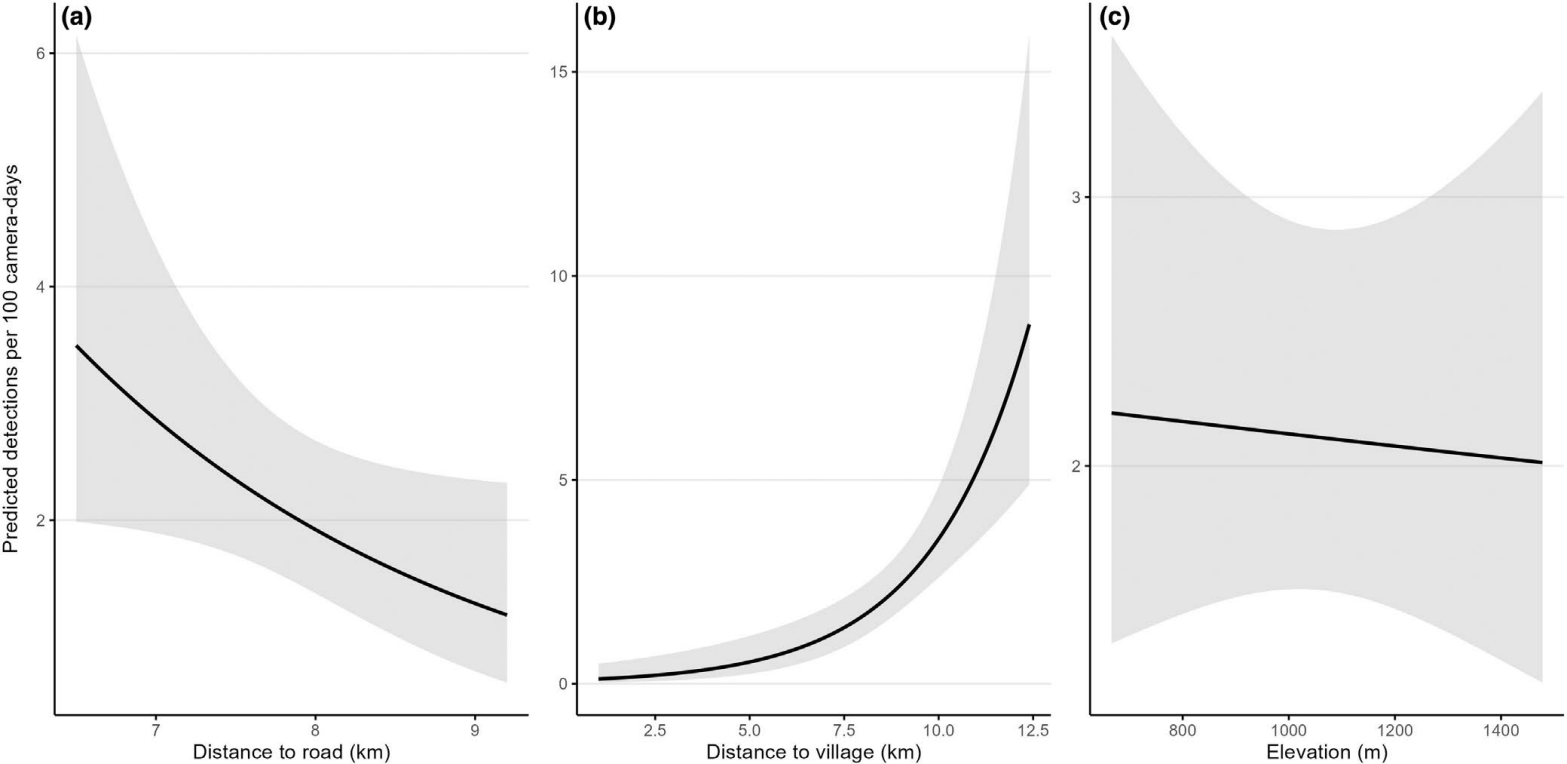
Relationship between the number of independent detection records of brown bears per camera trap in the study area in relation to (a) distance to the nearest road (km), (b) distance to the nearest village (km), and (c) elevation of the camera traps (m). Trend lines are from the generalized linear models.

We detected a total of 14 different species across 9 cameras which included independent detection records with up to 133 detections of brown bears, 126 for moose, 160 for red deer, and 368 for roe deer (Table 2). In addition, our records of nontarget species included 2 musk deer, 12 wild boar, 1 lynx, 1 wolf, 11 wolverine, 41 red fox, 11 Asian badger, 29 sable, 2 western capercaillie ( *Tetrao urogallus* ), and 4 mountain hare ( *Lepus timidus* ) (Table 2; Figure S1). The earliest and latest annual detection dates for brown bears were 1 April (in 2020) and 29 October (in 2019) for a 7 month period of seasonal activity (Figure 3). Brown bear detections were highest during May/June but otherwise remained relatively stable during the rest of the active period (Figure 3). Moose and maral detections were relatively steady throughout the year, while roe deer were detected primarily in the summer months (Figure 3). Bears appeared to be more active during the morning and evening, while moose and roe deer were more active during dawn and dusk (Figure 4). Brown bear detection rates were significantly related to proximity to human infrastructure. Detection rates decreased with increasing distance to the nearest road (*β* = −0.40 ± 0.20 SE, *z* = −1.99, *p* = 0.046), indicating higher detection rates closer to roads. In contrast, detection rates increased with distance from the nearest village (*β* = 0.38 ± 0.09 SE, *z* = 4.42, *p* < 0.001), suggesting lower bear activity or detectability near human settlements. Elevation had no detectable effect on brown bear detections within the study area (*β* = −0.0001 ± 0.0005 SE, *z* = −0.22, *p* = 0.83; Figure 5). The model explained a moderate proportion of variation in detection rates (McFadden’s pseudo‐R² = 0.24).

**TABLE 2:** Total number of detections per active camera trap for each species observed over the 5‐year study period (2019–2023) in the Altai Mountain Region study area in northeastern Kazakhstan.

	KCH01	KCH02	KCH03	KCH04	KCH05	KCH06	KCH07	KCH08	KCH09	KCH10
Brown bear	5	14	37	3	13	28	30	0	1	2
Moose	4	17	14	0	0	0	27	0	58	6
Caspian red deer	9	21	22	4	24	11	10	0	45	14
Siberian roe deer	19	6	31	17	34	30	84	0	128	19
Musk deer	0	2	0	0	0	0	0	0	0	0
Wild boar	3	3	4	0	0	0	1	0	0	1
Lynx	1	0	0	0	0	0	0	0	0	0
Wolf	1	0	0	0	0	0	0	0	0	0
Wolverine	0	2	0	0	0	0	6	0	3	0
Red fox	10	1	0	8	0	1	12	0	9	0
Siberian badger	2	0	1	0	0	0	8	0	0	0
Sable	4	1	5	0	0	1	16	0	2	0
Western capercaillie	2	0	0	0	0	0	0	0	0	0
Mountain hare	1	0	0	0	0	0	3	0	0	0

## 4 Discussion

Here, we present results from the first camera trapping operation designed to detect brown bears in the Altai Mountain Region of Kazakhstan. We detected a total of 14 species including brown bears, ungulates including potential prey species such as moose, Caspian red deer, and Siberian roe deer, and other carnivores during a 5 year monitoring program. Similar to other study systems, our results suggest that brown bears in the Atlai mountains were active for a 7 month period between early April and late October, which likely corresponds with approximate den exit in spring and den entry in fall (González‐Bernardo et al. 2020). Annual bear activity peaked in May/June, which coincides with both mating season when bears move further distances in search of mates (Dahle and Swenson 2003; Mattisson et al. 2019) as well as the season when bears search for newborn ungulate calves (Griffin et al. 2011; Rauset et al. 2012).

Young calves of moose and red deer are a common prey item for brown bears in many ecosystems (Griffin et al. 2011, Rauset et al. 2012), while roe deer are also preyed upon although less commonly (Blanco et al. 2011). Musk deer and wild boar are other potential prey for bears, but their detection rates were low in the study area. Moose activity appeared relatively stable throughout the year, whereas red deer activity showed a peak in September/October, which corresponds to the timing of rut/mating, as observed in other systems including nearby in Russia (Rusin et al. 2021). Roe deer were mostly only detected during the summer months (~May–October), which is likely related to seasonal migration of roe deer down to lower elevations during winter. Most ungulate species were not detected during mid‐winter, possibly because the camera traps were placed at relatively high elevations in the mountain areas (mean = 1046 m).

Our camera trap data revealed that bears were most active during the mornings and evenings, but were also regularly detected during the main part of the day. Our findings were unexpected because bears are commonly considered to be diurnal species. The diel patterns of activity could have been related to seasonality which is known to alter bear activity patterns (Ordiz et al. 2017). The diel activity patterns of the Siberian roe deer were consistent with previous studies by (Mori et al. 2021) who reported that roe deer in Mongolia were most active in between ~6:00–10:00 and ~19:00–21:00.

Our pilot study suggests that brown bears in the Altai Mountain Region were somewhat more likely to be detected on camera traps that were further away from villages and roads (Figure 5). Avoidance of human settlements and activities has been observed in multiple other ecosystems in both North America and Europe (e.g., Johnson et al. 2005; Preatoni et al. 2005; Corradini et al. 2024; Hertel et al. 2025). For example, brown bears in Scandinavia avoid high human activity and infrastructure including cities, towns, cabin developments, houses, recreational areas, and roads (Moe et al. 2007; Nellemann et al. 2007; Leclerc et al. 2016; Milleret et al. 2018), as well as encounters with people (Ordiz et al. 2011). Bears become more stressed when they are near areas of high human activity, such as settlements (Støen et al. 2015), which can result in a range of behavioral adaptations to avoid people, including shifting habitat use, altered movement patterns, or changes in circadian rhythms (Ordiz et al. 2014, 2017; Hertel et al. 2025).

At a regional scale, bears in the Altai Mountain Region of Kazakhstan probably avoid both areas of human activity and people, but the extent of habitat loss due to behavioral avoidance remains unknown. Individual bears that become food conditioned or habituated may show increased use of habitats near settlements or agricultural areas, which can lead to increased risk of conflicts with humans (Can et al. 2014; Bautista et al. 2017). Rates of human‐bear conflicts have been increasing in the Altai Mountain Region and a better understanding of the mechanisms underlying the increase is essential for building a sustainable regional plan for adaptive bear management. As of today, there is little structured management for brown bears in central Asia.

Our study was limited by three methodological issues which provide valuable lessons for future monitoring efforts in Kazakhstan. First, the small sample size of 10 cameras limited the spatial resolution of the monitoring program which ultimately affected our ability to model the detection data. For example, the low detection rate across all sites likely reflects the cryptic nature of bears and the limited number of sampling occasions per site. Second, the relatively confined study area (15 km^2^) restricted the generalizability of the findings to other parts of the Altai Mountain Region. The limitations highlight the importance of scaling up both the number of deployment sites and the geographic extent in future studies to enhance the accuracy and applicability of occupancy models. Last, the scope and design of our pilot project was limited by financial, logistical, and staffing resources; limited funding was available for the project and the field study was conducted by one PhD student who lived 12 h away from the field site.

There is limited national funding for monitoring brown bear populations across Kazakhstan, including the Altai Mountain Region. Funding shortfalls require development of an affordable, relatively passive, and reliable method for monitoring the population in the future. Researchers and managers in Kazakhstan can use the lessons learned from this pilot project to approach government funding agencies once they have a well‐developed plan for future monitoring. Cultural differences and local community engagement are also critical, as successful monitoring requires collaboration and respect for cultural practices and traditional knowledge of local people. For example, researchers need to be aware of the use of technology in wildlife monitoring as when done incorrectly, it can lead to division between conservation organizations and local communities (Shrestha and Lapeyre 2018). Future assessments in the area should continue to try and estimate occupancy at a broader scale across potential bear range in the Altai Mountain Region, as well as attempt to provide sufficient data for a feasible estimate of the local bear population size and the causes of bear‐human conflict.

The ultimate goal is to design a broader study that can deploy cameras in a more random and widespread way, while incorporating the use of local knowledge on where to deploy them and to monitor and upkeep the camera traps. Improvements to study design will make the project more cost‐effective and sustainable over a longer term. Our knowledge about the status of large carnivore populations remains relatively biased towards wealthier nations (Sheil 2001). It is important to support knowledge generation in developing countries to have a more complete understanding of our umbrella and keystone species for monitoring the status of the world's ecosystems. Furthermore, local knowledge will help facilitate management, tolerance, and conservation of predator species at the local scale, which can be scaled up to global patterns (Getz et al. 1999).

## Author Contributions

**Sanzhar Kantarbayev:** conceptualization (lead), data curation (lead), formal analysis (supporting), funding acquisition (lead), investigation (equal), methodology (lead), visualization (equal), writing – original draft (equal), writing – review and editing (lead). **Aimee Tallian:** conceptualization (equal), data curation (supporting), formal analysis (supporting), funding acquisition (supporting), investigation (supporting), methodology (supporting), visualization (supporting), writing – original draft (lead), writing – review and editing (equal). **Matthew Grainger:** conceptualization (equal), data curation (supporting), formal analysis (lead), funding acquisition (supporting), investigation (equal), methodology (lead), visualization (lead), writing – original draft (equal), writing – review and editing (equal). **Brett K. Sandercock:** conceptualization (equal), data curation (supporting), formal analysis (equal), funding acquisition (supporting), investigation (equal), methodology (supporting), visualization (equal), writing – original draft (supporting), writing – review and editing (supporting). **Alexander Kopatz:** conceptualization (lead), data curation (supporting), formal analysis (supporting), funding acquisition (equal), investigation (equal), methodology (supporting), visualization (supporting), writing – original draft (equal), writing – review and editing (equal).

## Funding

Our field project was funded by the International Bear Association Research & Conservation Grant Program. Sanzhar Kantarbayev's PhD work was funded by The State Educational Order for the Training of Specialists with Higher Education in Educational Institutions, funded from the republican budget under the Decree of the Government of the Republic of Kazakhstan (No. 199, dated April 16, 2018). This work was carried out as part of the projects of the Institute of Zoology of the Republic of Kazakhstan titled as «BR18574062 Assessment of biological resources of the Kazakh part of the transboundary Irtysh basin in the context of climate change». The Norwegian Institute for Nature Research received financial support from the Research Council of Norway (Project no. 160022/F40).

## Conflicts of Interest

The authors declare no conflicts of interest.

## Supporting information


**Figure S1:** Total number of detections per month per active camera trap in active cameras plotted for nontarget species that had > 3 detections including badger, brown bear, red fox, moose, red deer, roe deer, sable, wild boar, and wolverine in the Atlai mountains of Kazakhstan, 2019 to 2023.

## Data Availability

All data and code used in the manuscript are available at https://github.com/DrMattG/Sanzhar_Khazakstan/tree/main and https://doi.org/10.5281/zenodo.17986506.
