# Supplementary figures and images for: Monitoring Brown Bears in Kazakhstan: A Pilot Study from the Altai Mountain Region
Source: Ecol Evol. 2026 Mar 18;16(3):e73272. doi: 10.1002/ece3.73272 (PMC13093707; doi:10.1002/ece3.73272)

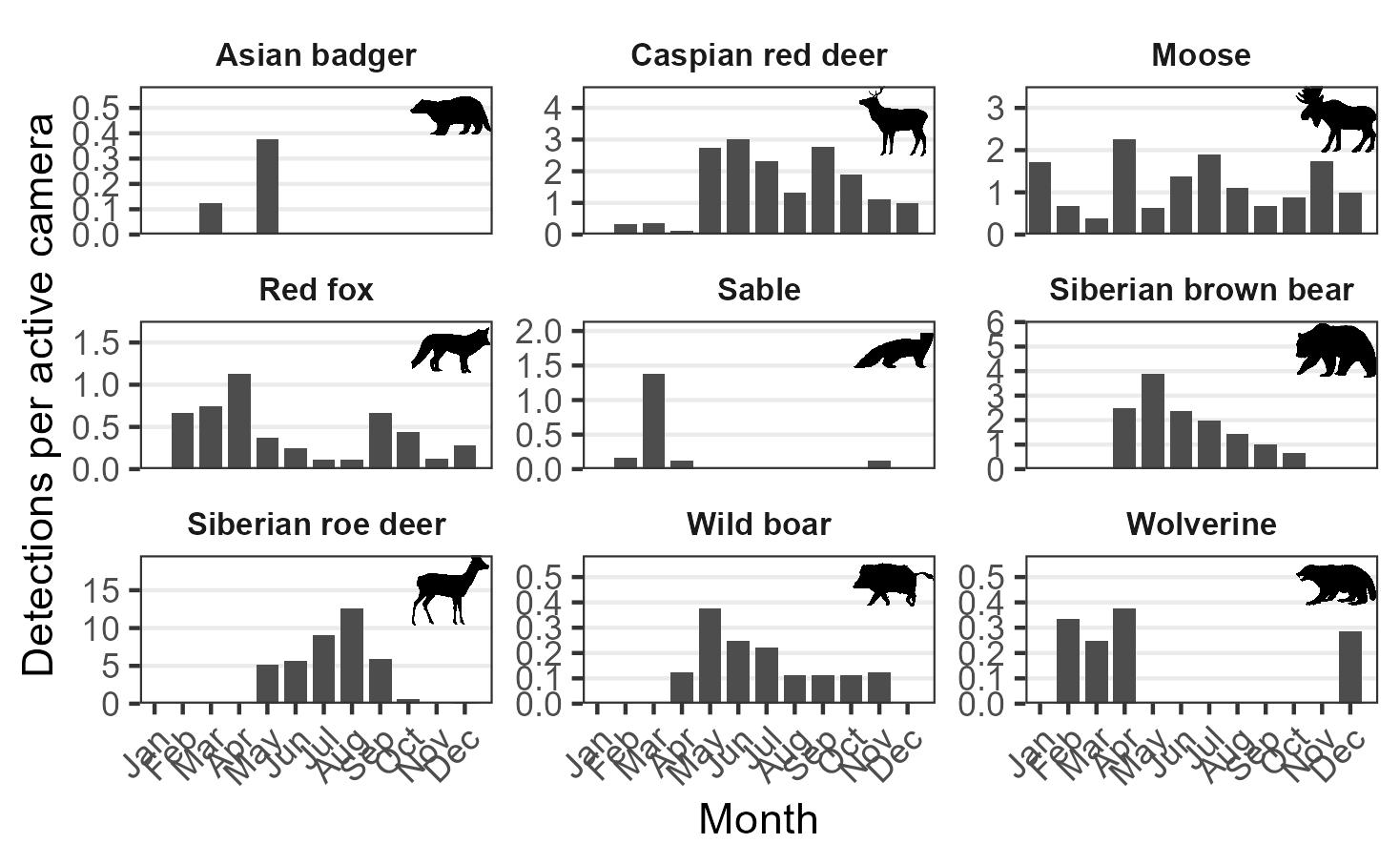

Supplement: Supplementary file 1 — Figure S1: Total number of detections per month per active camera trap in active cameras plotted for nontarget species that had > 3 detections including badger, brown bear, red fox, moose, red deer, roe deer, sable, wild boar, and wolverine in the Atlai mountains of Kazakhstan, 2019 to 2023. [file ECE3-16-e73272-s001.png]
